# *SNiPA*: an interactive, genetic variant-centered annotation browser

**DOI:** 10.1093/bioinformatics/btu779

**Published:** 2014-11-26

**Authors:** Matthias Arnold, Johannes Raffler, Arne Pfeufer, Karsten Suhre, Gabi Kastenmüller

**Affiliations:** ^1^Institute of Bioinformatics and Systems Biology, Helmholtz Zentrum München – German Research Center for Environmental Health, Ingolstädter Landstraße 1, 85764 Neuherberg, Germany and ^2^Department of Physiology and Biophysics, Weill Cornell Medical College in Qatar, Education City, Qatar Foundation, Doha, Qatar

## Abstract

**Motivation:** Linking genes and functional information to genetic variants identified by association studies remains difficult. Resources containing extensive genomic annotations are available but often not fully utilized due to heterogeneous data formats. To enhance their accessibility, we integrated many annotation datasets into a user-friendly webserver.

**Availability and implementation:** http://www.snipa.org/

**Contact:** g.kastenmueller@helmholtz-muenchen.de

**Supplementary information:** Supplementary data are available at *Bioinformatics* online.

## 1 Introduction

Genome-wide association studies (GWAS) and next-generation sequencing (NGS) are performed routinely to identify genetic variants and novel genes implicated in both common and rare human diseases. A key step in translating results from such studies into a better understanding of molecular disease mechanisms and, ultimately, into clinical applications, is the prioritization of potentially functional variants that may be active *in vivo*. To this end, comprehensive collection and evaluation of existing functional annotation from genetic, informatics and experimental resources is essential (MacArthur *et al.*, 2014). This comprises the integration of data and knowledge across multiple levels including the variant, the gene and the chromatin level.

Several large resources (Ensembl, UCSC, NCBI, etc.) aim at providing genome-wide genome-level annotation tracks from an extensive set of resources. However, retrieving statistical and functional annotation relevant at the single nucleotide level remains difficult. For instance, common genome browsers often display single nucleotide variants (SNVs) as thin bars that trail away in the wealth of other annotation tracks and are even less prepared to display statistics such as linkage disequilibrium (LD) relationships between variants. This limits visual distinction of relevant variants from those without relevant annotations and leaves the complex task of aggregating position-based data to the researcher. Variant-centered resources, on the other hand, typically concentrate on specific types of data such as amino acid changes (Adzhubei *et al.*, 2010; Kumar *et al.*, 2009), expression quantitative trait loci (eQTLs) (GTEx Consortium, 2013; Xia *et al.*, 2012), trait associations (Beck *et al.*, 2014; Hindorff *et al.*, 2009) or regulatory effect predictions (Boyle *et al.*, 2012). Moreover, these annotations are often presented in resource-specific data structures.

For individual inspection of single variants, both resource types are extremely valuable. However, for simultaneous processing of larger variant sets, collection and examination of annotations from different data sources quickly becomes cumbersome. This presents a major bottleneck in genome-wide scans of genetic influences on human traits since the collection of such evidences is the key to understanding the effects of phenotype-linked genetic variants.

**Fig. 1.**
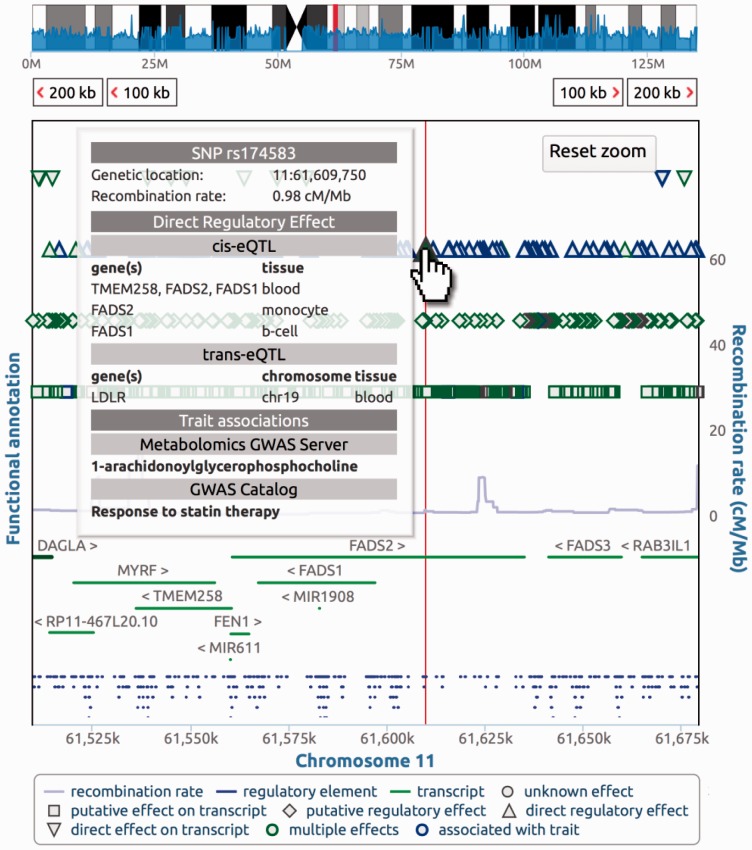
The *SNiPA* Variant Browser shows variants (top), genes (center) and regulatory regions (bottom). Top-level information is available in mouse-over tooltips for all plot elements as shown here for the query SNP *rs174583*. The example highlights the value of variant-centered accumulation of annotations: *rs174583* is associated with the concentration of a lipid metabolite as well as with the expression levels of two genes encoding enzymes involved in lipid metabolism (*FADS1/2*) and the gene coding for *LDL receptor*, a major regulator of cholesterol homeostasis. Furthermore, the variant was linked to the response to lipid lowering drugs (statins), which target *HMG-CoA reductase* regulated by the *LDL receptor*

Here we propose *SNiPA*, a web service offering variant-centered genome browsing and interactive visualization tools tailored for easy inspection of many variants in their locus context (Fig. 1).

## 2 Data and features

*SNiPA* includes a wide range of genome-level datasets contained in the Ensembl database (Flicek *et al.*, 2014) as an established backbone of annotations for the human genome. We combine this backbone with numerous variant-specific annotations taken from published datasets. Thus, *SNiPA* covers information ranging from regulatory elements, over gene annotations to variant annotations and associations (Table 1; Supplementary Text S1). *SNiPA* contains annotations for all bi-allelic variants in phase 3 version 5 of the 1000 genomes project (1000 Genomes Project Consortium *et al.*, 2012) and provides pre-calculated LD-data for *r*^2 ^≥ 0.1 for all super populations (African, American, South and East Asian, European). We use the Ensembl VEP tool (McLaren *et al.*, 2010) for primary effect prediction of SNVs. Additional position-based data is included in the VEP prediction as custom annotation files. For other annotations, we wrote a Perl module to extend the output provided by VEP (Table 1; Supplementary Text S1).

**Table 1.** Annotation data compiled in *SNiPA*

Entity type	Data type	*N*_Entries_^a^	*N*_Sources_^b^
Variant	*cis*-eQTL associations	919 860	8
	*trans*-eQTL associations	17 891	6
	Trait associations	245 333	9
	Conservation and deleteriousness scores	genome-wide	4
Gene	Trait annotations	3 752	3
Regulatory elements	microRNA target sites	606 408	5
	Promoters	106 169	2
	Enhancers	455 800	2
	ENCODE feature clusters	406 632	1

^a^Entries are unified w.r.t. the entities given in the first column, i.e. numbers listed are counts of annotated entities (e.g. variants).

^b^Details and references for all included datasets are described in Supplementary Text S1.

*SNiPA* provides user-friendly starting points for annotating individual SNVs as well as sets of SNVs, LD blocks or genetic regions of interest. We have implemented several entry points to access the data: (i) a variant-centered implementation of a genome browser (‘Variant Browser’); (ii) ‘Association Maps’ for browsing through GWAS results; (iii) an interface for batch retrieval of variant annotations via ID-list, gene ID or genomic coordinates (‘Variant Annotation’); (iv) a combined listing of annotations across a set of variants within LD blocks or chromosomal regions (‘Block Annotation’); (v) ‘Regional Association Plot’ and ‘Linkage Disequilibrium Plot’ (Diabetes Genetics Initiative of Broad Institute of Harvard *et al.*, 2007) that combine publication-ready plotting of association results and LD values, respectively, with the interactive interface of the ‘Variant Browser’; (vi) ‘Proxy Search’ and ‘Pairwise LD’ that allow querying precalculated LD values augmented with variant annotations. *SNiPA* enables the user to download condensed annotation data in tabular format for further off-line processing. Detailed descriptions of *SNiPA* modules are available in the online documentation and Supplementary Text S1.

The complex information contained in *SNiPA* is organized in a clear, comprehensive and informative structure extending effect categories contained in the Sequence Ontology (Eilbeck *et al.*, 2005) (Supplementary Text S1). For instance, variant annotations are presented as ‘*SNiPA*cards’ grouping information into semantic sections. All annotations are linked to their primary sources and to the Ensembl genome browser.

## 3 Conclusion

Mechanistic characterization of variants identified by genetic studies is the key to understanding molecular disease mechanisms. *SNiPA* combines a comprehensive set of genomic annotations with a genetic variant-based genome browser to simplify the task of variant annotation. *SNiPA* as well as all underlying data is freely available to the scientific community (commercial use may be limited by third-party constraints) and will be automatically updated following the Ensembl releases.

## Funding

This work was supported by the Helmholtz Portfolio theme ‘Metabolic Dysfunction and common disease’ and by the research project Greifswald Approach to Individualized Medicine (GANI_MED) (BMBF: 03IS2061A). K.S. is supported by Biomedical Research Program funds at Weill Cornell Medical College in Qatar, a program funded by the Qatar Foundation.

*Conflict of Interest*: none declared.

## Supplementary Material

Supplementary Data
