# Supplementary material for: SNiPA: an interactive, genetic variant-centered annotation browser
Source: Bioinformatics. 2014 Nov 26;31(8):1334–6. doi: 10.1093/bioinformatics/btu779 (PMC4393511; doi:10.1093/bioinformatics/btu779)
Supplement: Supplementary Data [file supp_31_8_1334__index.html]

SNiPA: an interactive, genetic variant-centered annotation browser — SNiPA: an interactive, genetic variant-centered annotation browser — SNiPA: an interactive, genetic variant-centered annotation browser — Supplementary Data 

# *SNiPA*: an interactive, genetic variant-centered annotation browser

## Supplementary Data

files

**Files in this Data Supplement:**

- Supplementary Data - pdf file
