# Supplementary material for: SNiPA: an interactive, genetic variant-centered annotation browser
Source: Bioinformatics. 2014 Nov 26;31(8):1334–6. doi: 10.1093/bioinformatics/btu779 (PMC4393511; doi:10.1093/bioinformatics/btu779)
Supplement: Supplementary Data [file supp_btu779_AppNote_SNiPA_rev_20141114_Supplementary_Text.pdf]

Supplementary Text to

SNiPA: an interactive, genetic variant-centered annotation browser

Matthias Arnold[1,*], Johannes Raffler[1,*], Arne Pfeufer[1], Karsten Suhre[1,2] , Gabi Kastenmüller[1,†]

Affiliations:

[1] Institute of Bioinformatics and Systems Biology, Helmholtz Zentrum München – German Research Center for Environmental Health, Ingolstädter Landstraße 1, D-85764 Neuherberg, Germany

[2] Department of Physiology and Biophysics, Weill Cornell Medical College in Qatar, Education City, Qatar Foundation, Doha, Qatar

[*] These authors contributed equally.

[†] Correspondence to: g.kastenmueller@helmholtz-muenchen.de (Gabi Kastenmüller).

Content

1 Contained data 2
1.1 Ensembl 2
1.2 Variant set 2
1.3 Conservation scores: phyloP, phastCons and GERP++ 2
1.4 Combined annotation dependent depletion (CADD) 3
1.5 Thurman et al. – promoters & distal enhancers/repressors 3
1.6 FANTOM5 – expressed promoters & enhancers/repressors 3
1.7 StarBase v2.0: miRNA target sites 3
1.8 eQTL data 3
1.9 Phenotype data 5
2 *SNiPA* features 6
2.1 General remarks 6
2.2 SNiPA effect categories 6
2.3 SNiPA modules 7
2.4 Updates / new releases 9
2.5 Documentation and feedback 9
3 References 10

Supplementary Text 1

1 Contained data

1.1 Ensembl

SNiPA makes extensive use of the Ensembl database (currently version 77) [16]. For genome-annotation we downloaded GENCODE gene data (including OMIM and DECIPHER annotations), regulatory feature clusters and regulatory motif data as well as linked information from the public MySQL database. The *SNiPA* database contains a total count of 59,006 genes with 206,671 associated transcripts and 99,121 protein products and 406,632 regulatory feature clusters, some of which are associated with transcription factor binding motifs. We also used many of the variant annotations as they are provided with the Variant Effect Predictor (VEP) [3] annotation. In addition, trait annotations and associations from OMIM [25], HGMD [22], UniProt [26], dbGaP [23] and ClinVar [24] were fetched from the public MySQL database. Details are given in the tables below.

1.2 Variant set

SNiPA annotates all bi-allelic single nucleotide variants contained in the 1000 Genomes Project phase 1 version 3 and phase 3 version 5 dataset [21]. For each super-population (AFR, AMR, ASN, EUR in phase 1; AFR, AMR, EAS, EUR, SAS in phase 3), linkage disequilibrium data for an $r^2 \geq 0.1$ are pre-calculated. The variant counts for the single super-populations are:

Phase 1 version 3:

African (AFR):	25,837,142
American (AMR):	20,097,916
Asian (ASN):	15,012,236
European (EUR):	17,361,202

Phase 3 version 5:

African (AFR):	39,581,182
American (AMR):	26,474,088
East Asian (EAS):	22,128,163
European (EUR):	22,541,970
South Asian (SAS):	24,854,259

1.3 Conservation scores: phyloP, phastCons and GERP++

Positional phyloP- as well as phastCons-100way-alignment PHAST conservation scores [1] in bigWig format were retrieved from
http://hgdownload.cse.ucsc.edu/goldenPath/hg19/phyloP100way/hg19.100way.phyloP100way.bw
and
http://hgdownload.cse.ucsc.edu/goldenPath/hg19/phastCons100way/hg19.100way.phastCons.bw.
Further information on assemblies used in the 100way alignment can be obtained at
http://hgdownload.cse.ucsc.edu/goldenPath/hg19/phyloP100way/. GERP++ positional RS (“rejected substitutions”) scores [2] were downloaded at

http://hgdownload.cse.ucsc.edu/gbdb/hg19/bbi/All_hg19_RS.bw. The three bigWig files were integrated into VEP annotation as custom annotation files. For VEP to be able to process bigWig files, we downloaded the bigWigToWig program provided by the University of California Santa Cruz [4].

1.4 Combined annotation dependent depletion (CADD)

Kircher et al. provide an annotation-aided score for genotype pathogenicity called CADD [5]. CADD-Scores for 1000 Genomes genotypes were obtained from http://cadd.gs.washington.edu/download. The downloaded file was parsed into one compressed Tabix-ready [6] file per chromosome (autosomes and X-chromosome) in General Feature Format (GFF, http://www.sanger.ac.uk/resources/software/gff/spec.html), Tabix-indexed and included in VEP annotation as custom annotation files. We used the PHRED-like transformation of the C score for variant annotation.

1.5 Thurman et al. – promoters & distal enhancers/repressors

In essence, Thurman et al. [7] used DNaseI hypersensitive sites (DHSs) and mapped them to transcription start sites (TSSs) of human transcripts. Accessible DHSs in proximity to the TSSs are classified as promoters. The accessibility patterns of more distal DHSs have been correlated with the accessibility patterns of promoters and are thus linked to the genes thought to be regulated by DHSs proximal to a TSS. After data processing, we obtained 412,798 distal elements (enhancers) and 23,749 promoters.

1.6 FANTOM5 – expressed promoters & enhancers/repressors

Two papers of the FANTOM5 consortium [8,9] describe the properties, location and transcript associations of expressed regulatory elements (promoters and enhancers). These datasets are provided at http://fantom.gsc.riken.jp/data/ and http://enhancer.binf.ku.dk/, respectively. After data processing, we included 82,420 expressed promoters and 43,002 expressed enhancers and their links to human transcripts in *SNiPA*.

1.7 StarBase v2.0: miRNA target sites

miRNA target sites located in RNA-binding protein (RBP) binding sites were obtained at the starBase v2.0 database (http://starbase.sysu.edu.cn/, released 09/2013, accessed 16/01/2014) [10]. We included target predictions from five prediction tools at positions that are located in experimentally identified regions bound by RBPs (n= 606,408). The downloaded file was parsed into one compressed Tabix-ready [6] file per chromosome (autosomes and X-chromosome) in General Feature Format (GFF, http://www.sanger.ac.uk/resources/software/gff/spec.html), Tabix-indexed and included in VEP annotation as custom annotation files.

1.8 eQTL data

Zeller et al., 2010 - monocytes

Zeller et al. investigated *cis*- and *trans*- associations of expression traits with >675,000 SNPs (Affymetrix SNP Array 6.0) in human monocytes from 1,490 unrelated individuals using the Illumina Human HT-12 v3 BeadChip. A SQLite database dump containing the association results is provided by the authors at http://genecanvas.ecgene.net/uploads/ForReview/ghs_probe_express030510.zip.

This database comprises imputed association data on >2 Mio. SNPs. Following the protocol in [11] associations were filtered for genome-wide significance ($P>5.78x10^{-12}$). This filtered set was intersected with Kruskall-Wallis (KW) test results and filtered to feature a KW $P<10^{-10}$ as described by Zeller et al. [11]. SNPs were then split into *cis-/trans*-associations via distance to their associated expression target (up to 1MB apart: *cis*, else: *trans*).

Multiple tissue human expression resource (MuTHER) – LCL, adipose and skin tissue

The MuTHER Consortium collected samples from 856 female twins of the TwinsUK resource in three tissues (LCL, adipose tissue, skin tissue) [12]. *cis*-eQTL associations comprising >2 Mio. SNPs were calculated using the Illumina Human HT-12 v3 BeadChip. Results files were retrieved from http://www.muther.ac.uk/Data.html and subjected to P-value filters as described in [12] ($P_{lcl}<7.8x10^{-5}$, $P_{adipose}<5x10^{-5}$, $P_{skin}<3.8x10^{-5}$) corresponding to a per-tissue false discovery rate (FDR) of 1%.

Westra et al., 2013 – peripheral blood

Westra et al. performed a meta-analysis of eQTL associations in peripheral blood samples from 5,311 individuals [13]. Genotype data was imputed to HapMap2 CEU genotypes (>2 Mio. SNPs), expression data from different Illumina platforms (Human HT-12 v3, HT-12 v4, and H8 v2 BeadChips) were harmonized by mapping probe sequences to Human HT-12 v3 identifiers. Association data was obtained at http://genenetwork.nl/bloodeqtlbrowser/. Probes specified by Illumina array address IDs were mapped to Illumina probe IDs using the developer manifest file (http://www.illumina.com). *Cis*- and *trans*-associations were filtered to have $P<1.31x10^{-4}$ and $P<5.12x10^{-7}$, respectively, corresponding to an FDR of 5%. In this study, eQTLs located less than 250 KB away from the probe midpoint are defined as *cis* while eQTLs more than 5 MB apart from the probe are defined as *trans* [13].

Fairfax et al., 2012 – B-cells and monocytes

Fairfax et al. investigated genotype associations with expression data from B-cells and monocytes from 288 individuals. For >600,000 SNPs *cis*- (<=2.5 MB away from the probe) and *trans*-associations were determined at permutation (n=1,000) $P<1x10^{-3}$ and Bonferroni-corrected $P<1x10^{-11}$, respectively. All significant associations from the online supplement [14] were mapped to Illumina HumanHT-12 v4 probes using the genomic coordinates provided in the supplemental files to obtain an up-to-date mapping to the corresponding genes. For this, hg18/NCBI36 coordinates had to be converted to hg19/GRCh37 coordinates using the UCSC liftOver tool [15]. Probe mapping data was retrieved from the Ensembl public SQL database [16].

seeQTL database – LCL and brain

The seeQTL database [17] contains several eQTL association datasets. Most of these are based on samples from individuals contained in the HapMap populations. On the data website of the seeQTL browser (http://www.bios.unc.edu/research/genomic_software/seeQTL/data_source), Xia et al. provide a meta-analysis association set on all HapMap-based studies which were included in *SNiPA*. In addition, association data from an eQTL study on human brain samples (Myers et al. [18]) in the same file format is available and was also included.

Dixon et al., 2007 - LCL

Dixon et al. investigated genotype associations with expression data (using Affymetrix HG-U133 Plus 2.0 chip) from LCL cell lines of 400 individuals [19]. The threshold for genome-wide significance was set

to be a LOD score >6.076 (equivalent to an FDR of 5%). Significant associations were extracted from the online supplement [19]. Associations with probes mapping to multiple locations in the genomes where removed (n=3,309). Associations were defined as *trans* if SNPs are located more than 1 MB apart from the probe center, and *cis* else.

Innocenti et al., 2011 - hepatocytes

Innocenti et al. investigated genotype associations with expression data (using Agilent 4x44K arrays) from liver tissue of 266 individuals [20]. The threshold for genome-wide significance was described to be a Bayes factor of >5. We downloaded significant *cis*-associations from the online supplement [20]. *SNiPA* reports the P-values provided with the associations that, thus, may not always seem to be significant on a genome-wide level.

1.9 Phenotype data

In addition to the data obtained at Ensembl, we included the NHGRI GWAS Catalog and gene annotations from OrphaNet (details below).

Variant associations & annotations

Source	N (unique)	Reference
**HGMD**	35,326 (31,770)	PMID: 24077912 [22]
**dbGaP**	41,426 (28,824)	PMID: 17898773 [23]
**ClinVar**	89,522 (87,923)	PMID: 24234437 [24]
**OMIM variation**	9,595 (8,968)	http://omim.org/ [25]
**UniProt**	3,573 (3,366)	PMID: 24253303 [26]
**GWAS Catalog**	16,342 (15,343)	PMID: 19474294 [27]
**DrugBank 4.0**	179 (169)	PMID: 24203711 [28]

Gene associations

Source	N (unique)	Reference
**DECIPHER**	1,795 (1,795)	http://decipher.sanger.ac.uk/
**OMIM gene**	5,055 (5,051)	http://omim.org/ [25]
**OrphaNet**	5,684 (5,684)	http://orpha.net/ [29]

2 *SNiPA* features

2.1 General remarks

SNiPA is a variant-centered resource. There are only two additional annotation tracks available: a gene annotation track and a track consisting of regulatory elements. The latter are linked to Ensembl or to their primary sources, if available. The central content of *SNiPA* are the “*SNiPA* cards” containing the annotation of individual variants. As “*SNiPA* cards” are very detailed and thus cannot be compressed enough to be manageable when investigating large variant sets, we also provide two other displays of annotations: first, the Block Annotation which is basically a “*SNiPA* card” except that it merges the annotation of all variants specified by the user. And second, a tabular format that contains top-level annotations for the variants (one row per variant). These tables can be sorted and filtered by keywords and individual “*SNiPA* cards” can then directly be accessed. Tables (as CSV) and “*SNiPA* cards” (as PDF) can be downloaded for later use.

Dependent on the used *SNiPA* module, one of the following input types is required by the user: dbSNP rs-identifier(s), a gene identifier, or a chromosomal position. For convenience, we have collected various sets of gene identifiers (such as UniGene IDs, Entrez Gene IDs, HGNC gene symbols, and so on) which makes previous mapping to Ensembl gene IDs (the ID scheme used by *SNiPA*) unnecessary in most cases.

The approaches behind *SNiPA*’s modules are logically separated by design. However, it is often necessary to analyze variants from different points of view. To simplify that, we have implemented a global interface (“Variant clipboard”) that can be used to store variants of interest. The input forms of all *SNiPA* modules provide functionality to paste variants from the clipboard into the form. This enables, for instance, scrolling through the genome using the Variant Browser, selecting variants of interest, and afterwards switching to the Variant Annotation module (or the Block Annotation if all variants are located on the same chromosome) to retrieve the “*SNiPA* cards” for all variants at once.

2.2 SNiPA *effect categories*

SNiPA uses the VEP [3] for primary variant effect predictions. VEP provides Sequence Ontology (SO) [30] terms with its predictions that we have categorized into several groups. For all additional annotations that *SNiPA* provides, we also use SO terms internally (e.g. level_of_transcript_variant for eQTL associations) if possible. For all other variants (e.g. variants within RBP binding sites), we used custom terms but did this in an SO-like way (e.g. miRNA_target_site_variant). Details on SO terms can be accessed at the SO website (www.sequenceontology.org/). The effect categories in *SNiPA* are defined as follows (from most severe to least severe):

1. Category: "Direct transcript effect"
SO terms:
coding_sequence_variant, frameshift_variant, incomplete_terminal_codon_variant, initiator_codon_variant, mature_miRNA_variant, missense_variant, splice_acceptor_variant, splice_donor_variant, stop_gained, stop_lost

2. Category: "Direct regulatory effect"
SO terms:
level_of_transcript_variant

3. Category: "Putative regulatory effect"
SO terms:
regulatory_region_variant, TF_binding_site_variant, downstream_gene_variant (subcategory "Proximal to gene variant"), *upstream_gene_variant* (subcategory "Proximal to gene variant")

Non-SO terms:
miRNA_target_site_variant

4. Category: "Putative transcript effect"
SO terms:
3_prime_UTR_variant, 5_prime_UTR_variant, intron_variant, nc_transcript_variant, NMD_transcript_variant, non_coding_exon_variant, splice_region_variant, stop_retained_variant, synonymous_variant

5. Category "Unknown effect"
SO terms:
intergenic_variant

Annotations contained in "*SNiPA* cards" are grouped in the first four categories. In addition, there is another section holding information on trait annotations for variants and genes as well as one section on general information on the variant.

The categories are encoded in all visualizations via the symbol used for the single variants (symbol keys are always listed in legends).

2.3 SNiPA *modules*

Currently, there are eight modules that allow for retrieval of the data contained in *SNiPA.* In the following, we will shortly introduce them to emphasize their underlying concepts. Detailed usage instructions are given in the documentation section on the *SNiPA* website.

Variant Browser

The *SNiPA* variant browser is our version of a genome browser with a variant-centered point of view (Figure 1A). Our main focus here was to enable the user to visually assess how well the variants in a locus are characterized by evidences. To achieve that, variants are plotted according to their highest effect category (see *2.2 SNiPA effect categories*) meaning that the higher a variant is located in the plot, the more evidence exists for it to feature strong effects. Variants that are assigned to more than one effect category are highlighted in green, variants that have trait annotations available are highlighted in blue. Here, the symbols used for the variants and their location in the plot are redundant information. This is because the two other interactive plotting modules of *SNiPA* (LD plot (Figure 1B) and regional association plot) implement the interface of the browser and use other means of variant positioning, and there the used symbol is the only visual hint at the assigned effect categories.

The variant browser is intended to provide inspection of genomic loci without a background hypothesis. For these, other modules are better suited (see below).

An additional feature of the browser (and of all visualizations implementing the browser's interface) is that the display can be exported as vector image, PDF, or PNG.

Association Maps

To inspect variants or sets of variants that are associated with a specific trait (or a set of traits), we have implemented this module that allows for access to the data in *SNiPA* for variants with published associations (Figure 1D). "*SNiPA* cards" of the variants can be directly accessed from the karyogram. Furthermore, variants can be added to the Variant clipboard and then be input into other modules such as the linkage disequilibrium plot for an LD-based locus inspection, the LD-based block annotation to get a summary of annotations for all correlating variants, the proxy search to retrieve a table of these variants with or without dense annotations, or the variant browser for further inspection of flanking regions of the locus.

Variant Annotation

This module provides direct access to variant annotations contained in *SNiPA*. Given a user-specified list of rs-identifiers, *SNiPA* returns a list of "*SNiPA* cards".

Block Annotation

SNiPA's block annotation module enables retrieval of merged annotations of a set of variants that can be specified by four different ways: a list of rs-identifiers, one rs-identifier that is first used to obtain a list of correlating variants (user-specified LD-threshold), a gene identifier, or a chromosomal region. Currently, only variants located on the same chromosome can be processed by block annotation. The merged annotation can be used to characterize a whole locus and thus may also be useful for characterizing rare variants for which no annotations are available.

Regional Association Plot

This is the classical plot for visualizing association results (locus-based Manhattan plot). Input is a user-specified list of variant/association p-value pairs. Variants are plotted by their position on the x-axis and $-\log_{10}$(p-value) on the y-axis. In addition, variants are colored by their correlation with the sentinel variant (by default, this is the variant with the lowest p-value, but optionally it can also be specified by the user). This plot implements the interface of the variant browser, meaning that all functionalities of the variant browser are provided except for navigating to other loci.

Linkage Disequilibrium Plot

This plot is very useful for instance to inspect a published GWAS hit. It is common practice to select a single variant (e.g. the one with the lowest p-value) as published representative for an association signal. LD data can be used to reproduce the reported locus albeit there always will be differences as the study populations will not be perfectly resembled by 1000 genomes individuals. Input is a single rs-identifier. Variants are plotted by their position on the x-axis and their correlation (r^2) to the specified variant on the y-axis. This plot implements the interface of the variant browser, meaning that all functionalities of the variant browser are provided except for navigating to other loci. Instead, the plot can be updated by selecting any contained variant as locus representative.

Proxy Search

This module allows for tabular retrieval of variants in LD with input variants. Dense annotation of the resulting variant set is possible.

Pairwise LD

A common challenge of association studies is to find out if one locus contains more than one association signal. One possible (albeit not the optimal) approach to do so is to check the LD pattern of the variants contained in the locus which can be done using this module.

2.4 Updates / new releases

Like Ensembl, SNiPA updates will come in quarterly (or, if Ensembl updates are minor, semiannual) releases. Updates will include the incorporation of new 1000 genomes data releases (if available) as well as complete updates of the Ensembl-based datasets. Using our custom variant annotator, the updated information will be merged with the additional datasets in the SNiPA collection. As soon as all annotations used by SNiPA are available for the GRCh38 genome assembly, we will include this assembly into SNiPA. GRCh37 data will be retained in parallel for a reasonable time.

Release notes will be listed in a corresponding section on the SNiPA website.

2.5 Documentation and feedback

SNiPA is a new resource and thus we are dependent on input from external users with respect to improvements of the resource (such as inclusion of additional datasets), development of new modules, as well as the usefulness of the help texts in input forms or the documentation.

We have already created a rudimental FAQ-like documentation. However, we want to extend this as well as we are open for any suggestions for improvement of *SNiPA*. Therefore, we would be very thankful for all hints, bug reports, questions, and suggestions. These can be sent to either the corresponding author or directly to feedback@snipa.org.

3 References

1. Siepel, A. et al. Evolutionarily conserved elements in vertebrate, insect, worm, and yeast genomes. *Genome research* **15**, 1034-1050 (2005).
2. Davydov, E.V. et al. Identifying a high fraction of the human genome to be under selective constraint using GERP++. *PLoS computational biology* **6**, e1001025 (2010).
3. McLaren, W. et al. Deriving the consequences of genomic variants with the Ensembl API and SNP Effect Predictor. *Bioinformatics* **26**, 2069-2070 (2010).
4. Kent, W.J., Zweig, A.S., Barber, G., Hinrichs, A.S. & Karolchik, D. BigWig and BigBed: enabling browsing of large distributed datasets. *Bioinformatics* **26**, 2204-2207 (2010).
5. Kircher, M. et al. A general framework for estimating the relative pathogenicity of human genetic variants. *Nature genetics* **46**, 310-315 (2014).
6. Li, H. Tabix: fast retrieval of sequence features from generic TAB-delimited files. *Bioinformatics* **27**, 718-719 (2011).
7. Thurman, R.E. et al. The accessible chromatin landscape of the human genome. *Nature* **489**, 75-82 (2012).
8. Fantom Consortium and the Riken PMI and CLST (DGT) et al. A promoter-level mammalian expression atlas. *Nature* **507**, 462-470 (2014).
9. Andersson, R. et al. An atlas of active enhancers across human cell types and tissues. *Nature* **507**, 455-461 (2014).
10. Li, J.H., Liu, S., Zhou, H., Qu, L.H. & Yang, J.H. starBase v2.0: decoding miRNA-ceRNA, miRNA-ncRNA and protein-RNA interaction networks from large-scale CLIP-Seq data. *Nucleic acids research* **42**, D92-97 (2014).
11. Zeller, T. et al. Genetics and beyond--the transcriptome of human monocytes and disease susceptibility. *PloS one* **5**, e10693 (2010).
12. Grundberg, E. et al. Mapping cis- and trans-regulatory effects across multiple tissues in twins. *Nature genetics* **44**, 1084-1089 (2012).
13. Westra, H.J. et al. Systematic identification of trans eQTLs as putative drivers of known disease associations. *Nature genetics* **45**, 1238-1243 (2013).
14. Fairfax, B.P. et al. Genetics of gene expression in primary immune cells identifies cell type-specific master regulators and roles of HLA alleles. *Nature genetics* **44**, 502-510 (2012).
15. Rhead, B. et al. The UCSC Genome Browser database: update 2010. *Nucleic acids research* **38**, D613-619 (2010).
16. Flicek, P. et al. Ensembl 2014. *Nucleic acids research* **42**, D749-755 (2014).
17. Xia, K. et al. seeQTL: a searchable database for human eQTLs. *Bioinformatics* **28**, 451-452 (2012).
18. Myers, A.J. et al. A survey of genetic human cortical gene expression. *Nature genetics* **39**, 1494-1499 (2007).
19. Dixon, A.L. et al. A genome-wide association study of global gene expression. *Nature genetics* **39**, 1202-1207 (2007).
20. Innocenti, F. et al. Identification, replication, and functional fine-mapping of expression quantitative trait loci in primary human liver tissue. *PLoS genetics* **7**, e1002078 (2011).
21. 1000 Genomes Project Consortium et al. An integrated map of genetic variation from 1,092 human genomes. *Nature* **491**, 56-65 (2012).
22. Stenson, P.D. et al. The Human Gene Mutation Database: building a comprehensive mutation repository for clinical and molecular genetics, diagnostic testing and personalized genomic medicine. *Human genetics* **133**, 1-9 (2014).
23. Mailman, M.D. et al. The NCBI dbGaP database of genotypes and phenotypes. *Nature genetics* **39**, 1181-1186 (2007).
24. Landrum, M.J. et al. ClinVar: public archive of relationships among sequence variation and human phenotype. *Nucleic acids research* **42**, D980-985 (2014).

25. Online Mendelian Inheritance in Man (OMIM®) [http://omim.org/ - accessed: 02/27/2014] (McKusick-Nathans Institute of Genetic Medicine, Johns Hopkins University, Baltimore, MD; 1966-2014).
26. UniProt Consortium Activities at the Universal Protein Resource (UniProt). *Nucleic acids research* **42**, D191-198 (2014).
27. Hindorff, L.A. et al. Potential etiologic and functional implications of genome-wide association loci for human diseases and traits. *Proceedings of the National Academy of Sciences of the United States of America* **106**, 9362-9367 (2009).
28. Law, V. et al. DrugBank 4.0: shedding new light on drug metabolism. *Nucleic acids research* **42**, D1091-1097 (2014).
29. Orphanet encyclopedia, Edn. 03/2014 (http://orpha.net/).
30. Eilbeck, K. et al. The Sequence Ontology: a tool for the unification of genome annotations. *Genome biology* **6**, R44 (2005).
